# Prenatally Diagnosed Testicular Torsion: A Rare Condition That Causes Dilemma in Management

**DOI:** 10.1155/2021/8825763

**Published:** 2021-01-18

**Authors:** Michael Gerbo, Chad Crigger, Yasamin Samadi, Michael C. Ost, Osama Al-Omar

**Affiliations:** West Virginia University Department of Urology, Division Pediatric Urology, Suite 6300, Health Sciences Center, Morgantown, WV 26506, USA

## Abstract

**Background:**

Prenatal testicular torsion (PTT) is exceedingly rare in intrauterine development, often diagnosed at the time of birth and very rarely diagnosed in utero during routine gestational ultrasound. As a result, incidence is unknown, and there exists no consensus regarding the pathophysiology of this phenomenon nor universally recognized algorithms and guidelines regarding its diagnosis and management. *Case Presentation*. We present the case of an antenatally diagnosed torsion and our subsequent management which included ipsilateral orchiectomy and prophylactic contralateral orchiopexy via a scrotal approach.

**Conclusion:**

While controversy regarding surgical intervention in patients with unilateral PTT exists due to poor salvage rates—estimated to be less than 1%—the risk of anorchia is higher in affected patients due to limitations in the accuracy of detecting bilateral testicular involvement. Risk of misdiagnosis of bilaterality may lead to lasting sequelae such as infertility and devastating psychological consequences for affected patients, supporting the need for surgical exploration, as was performed in our case.

## 1. Introduction

Prenatal testicular torsion (PTT) is an exceedingly rare type of neonatal torsion that occurs during the intrauterine period. While the exact pathophysiology remains unknown, it is believed that a hostile intrauterine environment with increased stress—particularly seen in high risk deliveries—leads to extreme cremasteric tension and subsequent torsion [1]. True incidence rates are unknown as PTT is usually coincidentally discovered during routine prenatal ultrasound examination. Postnatally, time from diagnosis to treatment directly influences survival of the affected organ; however, there is little consensus on the management of PTT given salvage rates of the affected testicle are less than 1%, with estimates ranging from 0% to 5% [2,3].

Furthermore, complicating the controversy surrounding surgical intervention in PTT is the increased risk of anorchia in patients with unilateral PTT due to inherent limitations in clinical and radiographic diagnoses. Due to lack of universal diagnostic and management guidelines, treatment varies from conservative management to prompt surgical intervention. Furthermore, the decision of surgical intervention varies among physicians—ranging from ipsilateral orchiectomy of the affected testicle to ipsilateral orchiectomy with contralateral fixation and even preservation of the ipsilateral testis plus fixation of the contralateral testis when possible [2, 3]. In what follows, we present a case of unilateral PTT, diagnosed in utero, with a highlight on early radiologic diagnosis and prompt surgical management, aimed at preventing bilateral torsion events.

## 2. Case Presentation

**Figure 1:**
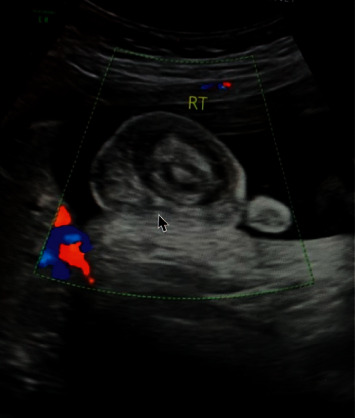
In utero fetal color Doppler ultrasonography demonstrating a sagittal view of right testicle at 36 weeks gestation, revealing a thickened scrotal wall (arrow) with reactive hydrocele surrounding a necrotic testicle.

A 30-year-old G2P1 female with a history of loop electrosurgical excision procedure (LEEP) and prior caesarean delivery was monitored in early pregnancy. She received four routine prenatal ultrasonography (US) at 8, 12, 19, and 28 weeks of gestation, during which time, the sonograms demonstrated a male fetus with no apparent anatomical anomalies. At 36 weeks, a prenatal US was performed due to inconsistent fetal size. Initial radiologic interpretation revealed a hemorrhagic incident with thrombosis of the right testicle (Figure 1). The right fetal scrotum appeared enlarged, while the left testicle appeared normal. These were new findings when compared to the US study from 28 weeks.

**Figure 2:**
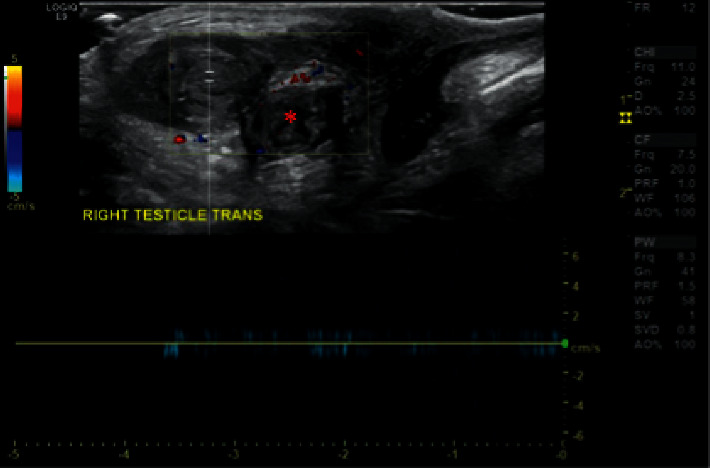
Sagittal view of immediate postnatal color Doppler ultrasonography demonstrating the absence of testicular blood flow and accompanying changes including “whirlpool” sign (asterisk).

The infant was born at an offsite facility via low, transverse caesarean delivery at 39 weeks of gestation, weighing 3640 g. Apgar scores were 8 and 9 at 1 and 5 minutes, respectively. At six hours of life, a scrotal US was again obtained due to concern for the right testicle. This showed an asymmetrically heterogeneous and small testicle. Color Doppler ultrasonography demonstrated absence of blood flow in the right testicle accompanied by chronic changes in the tissue consistent with a subacute or chronic testicular infarct (Figure 2).

**Figure 3:**
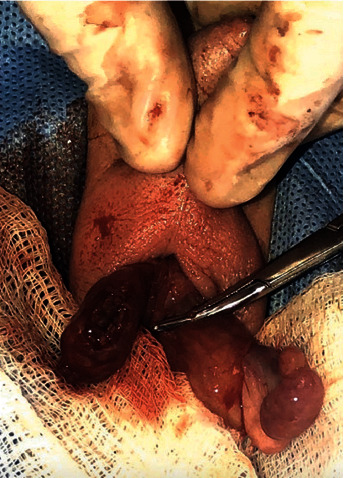
The incision of the scrotum along the raphe, revealing a discolored, edematous, and necrotic right testicle, with normal appearance of the left testicle. Orchiectomy of the right testicle was followed by contralateral orchiopexy.

The patient was then transferred to our facility. Upon arrival, physical exam at bedside was normal with no scrotal edema, erythema, or pain upon palpation. Repeat scrotal US revealed absence of blood flow to the right testicle in addition to accompanying chronic atrophic changes. Scrotal exploration via a midline incision was then performed which revealed right extravaginal testicular torsion with a necrotic right testicle. Subsequent simple orchiectomy was performed (Figure 3). The left testicle was found to be viable, and scrotal orchiopexy was performed to prevent contralateral torsion. The newborn recovered well postoperatively and was discharged home.

## 3. Discussion

Testicular torsion overall has a bimodal distribution with the highest incidence of torsion occurring after puberty, followed by the neonatal period. Intravaginal torsion occurs in the former, and torsion is almost exclusively extravaginal in the latter [4]. Of those torsions that occur in the perinatal period, only 20% is postnatal events. PTT is a rare and incidentally found insult with potentially devastating long-term effects. Though high-frequency color Doppler ultrasonography is the preferred imaging study, the results must be interpreted with caution as detection of blood flow is not always reliable in small prepubertal testes [5]. Others have fortunately found more reliable adjunct radiologic findings including “swirl sign” or “whirlpool sign” which describes the appearance of the twisted spermatic cord and has proven to be a better predictor of at least childhood testicular torsion when compared to Doppler ultrasonography in accurately predicting torsion in 96% of patients [6].

While imaging may aid in diagnosis, the clinical picture and presentation cannot be overlooked. Presenting signs and symptoms can vary widely further muddying the clinical picture. Neonatal torsion in the acute phase may present as an enlarged, hardened testicle often with apparent scrotal inflammation including edema and hyperemia. Other possible differential diagnoses that may mimic acute phase torsion including complete inguinal (scrotal) hernia, scrotal tumor, epididymoorchitis, and torsion of a testicular-epididymal appendage [7]. Chronic phase torsion is characterized by necrosis, decreased size and fibrosis, and even an echogenic “halo” on ultrasound, or calcium deposits [8]. Ecchymosis and discoloration can often be seen in either scenario or as the result of trauma from birth.

The lack of consensus and variability in management is perhaps the greatest dilemma urologists face when treating this condition. Due to the poor rate of salvaging a testicle in the perinatal period—as most of these represent irreversible intrauterine events—some argue that there is no advantage to early intervention and that surgery should be deferred until the risks of anesthesia and surgery are better optimized [1, 2]. Others argue that, although bilateral asynchronous torsion is rare, performing contralateral fixation within a reasonable time and not delaying are preferred given the risk of anorchia and its devastating effects, especially given the relative safety of prophylactic orchiopexy [9].

In their case series and eloquent meta-analysis, Melcer et al. utilized a different definition of prenatal torsion as that diagnosed in utero or immediately after birth upon newborn examination [10, 11]. Of their five cases of prenatal torsion, two were diagnosed antenatally. Both of these cases underwent urgent caesarean delivery, and one was managed with scrotal exploration while the other was managed conservatively. In the case that was explored, the affected testis was atrophic and nonsalvageable, and orchiectomy was performed with contralateral orchiopexy. For the case managed conservatively, follow-up ultrasonography at 3 months confirmed a completely atrophic testis. Based on the results of their study, they proposed the first algorithm for intervention in case of antenatally diagnosed torsion. In utero diagnosis of unilateral torsion alone is not strong enough of an indication to induce delivery unless there is clear evidence of an acute torsion seen by follow-up ultrasound in less than 24 hours, since salvage of the affected testicle is improbable. In all other cases, observation with possibility of intervention after birth is recommended. They propose that induced delivery with intervention is only indicated when synchronous bilateral torsion of the testes is suspected at >34 weeks gestation. Even if bilateral synchronous torsion is suspected <34 weeks gestation, an observational approach is advocated. This rare and hypothetical case of bilateral synchronous torsion diagnosed antenatally requiring intervention has also been proposed by others [12].

Surgical approaches vary too. Ipsilateral orchiectomy is not always advised as ipsilateral autotesticular atrophy or leaving an affected testicle in place due to the possibility of some, albeit small, endocrine function [2, 13]. While ipsilateral orchiectomy may be performed, contralateral orchiopexy rates vary and so too the same possible combinations via inguinal or scrotal approaches [14].

Our case represents a chronic phase extravaginal torsion diagnosed antenatally on routine obstetric ultrasonography. After conferring with the patient's parents, we performed urgent surgical exploration with ipsilateral orchiectomy and contralateral orchiopexy via a scrotal approach. While salvage of the ipsilateral affected testicle was not our intent, surgical intervention was pursued to ensure bilateral synchronous torsion was not present and to also eliminate the risk of anorchia.

## 4. Conclusions

Current management of PTT ranges from no intervention during the time of diagnosis to possibly even inducing delivery during intrauterine diagnosis and surgical intervention in the neonate. Therapeutic approach is made on a case-by-case basis, with a significant burden placed on weighing the risks and benefits of surgery in a neonate against future infertility, in addition to devastating physiologic, social, and psychiatric consequences for the patient. Further research studies regarding diagnostic intervention and identification of treatment outcomes and prognosis are essential to further the body of evidence to support the development of an evidence-based universal algorithm which would in turn facilitate the most efficacious diagnosis and management approach for this rare clinicopathologic condition.

## Data Availability

No data were used to support this study.

## References

[1] Kaye J. D., Levitt S. B., Friedman S. C., Franco I., Gitlin J., Palmer L. S. (2008). Neonatal torsion: a 14-year experience and proposed algorithm for management. *Journal of Urology*.

[2] Djahangirian O., Ouimet A., Saint-Vil D. (2010). Timing and surgical management of neonatal testicular torsions. *Journal of Pediatric Surgery*.

[3] Clarke M. J. H., Crocker S., Bartle D. G., Apsey J. (2018). Bilateral testicular torsion in a 36-week neonate. *Case Reports*.

[4] Weingarten J. L., Garofalo F. A., Cromie W. J. (1990). Bilateral synchronous neonatal torsion of spermatic cord. *Urology*.

[5] Atkinson G. O., Patrick L. E., Ball T. I., Stephenson C. A., Broecker B. H., Woodard J. R. (1992). The normal and abnormal scrotum in children: evaluation with color Doppler sonography. *American Journal of Roentgenology*.

[6] Kalfa N., Veyrac C., Lopez M. (2007). Multicenter assessment of ultrasound of the spermatic cord in children with acute scrotum. *Journal of Urology*.

[7] Mano R., Livne P. M., Nevo A., Sivan B., Ben-Meir D. (2013). Testicular torsion in the first year of life - characteristics and treatment outcome. *Urology*.

[8] Devesa R., Muñoz A., Torrents M., Comas C., Carrera J. M. (1998). Prenatal diagnosis of testicular torsion. *Ultrasound in Obstetrics and Gynecology*.

[9] Lopez R. N., Beasley S. W. (2012). Testicular torsion: potential pitfalls in its diagnosis and management. *Journal of Paediatrics and Child Health*.

[10] Melcer Y., Mendlovic S., Klin B. (2015). Fetal diagnosis of testicular torsion: what shall we tell the parents?. *Prenatal Diagnosis*.

[11] Herman A., Schvimer M., Tovbin J., Sandbank J., Bukovski I., Strauss S. (2002). Antenatal sonographic diagnosis of testicular torsion. *Ultrasound in Obstetrics and Gynecology*.

[12] Ryken T. C., Turner J. W., Haynes T. (1990). Bilateral testicular torsion in a pre-term neonate. *Journal of Urology*.

[13] Callewaert P. R. H., Van Kerrebroeck P. (2010). New insights into perinatal testicular torsion. *European Journal of Pediatrics*.

[14] Kylat R. (2017). Neonatal testicular torsion: is it time for consensus?. *Journal of Clinical Neonatogly*.

